# Using CRISPR/Cas9-Mediated *GLA* Gene Knockout as an In Vitro Drug Screening Model for Fabry Disease

**DOI:** 10.3390/ijms17122089

**Published:** 2016-12-13

**Authors:** Hui-Yung Song, Huai-Chih Chiang, Wei-Lien Tseng, Ping Wu, Chian-Shiu Chien, Hsin-Bang Leu, Yi-Ping Yang, Mong-Lien Wang, Yuh-Jyh Jong, Chung-Hsuan Chen, Wen-Chung Yu, Shih-Hwa Chiou

**Affiliations:** 1 Institute of Pharmacology, National Yang-Ming University, Taipei 11221, Taiwan; shy770307@gmail.com (H.-Y.S.); shchiou@vghtpe.gov.tw (S.-H.C.); 2 Department of Medical Research, Taipei Veterans General Hospital, Taipei 11217, Taiwan; skechiang@gmail.com (H.-C.C.); jan740709@gmail.com (W.-L.T.); Pingwu821006@gmail.com (P.W.); cschien6688@gmail.com (C.-S.C.); hbleu@vghtpe.gov.tw (H.-B.L.); molly0103@gmail.com (Y.-P.Y.); monglien@gmail.com (M.-L.W.); 3 School of Medicine, National Yang-Ming University, Taipei 11221, Taiwan; 4 Division of Cardiology & Department of Medicine, Taipei Veterans General Hospital, Taipei 11217, Taiwan; 5 Graduate Institute of Clinical Medicine, College of Medicine, Kaohsiung Medical University Hospital, Kaohsiung Medical University, Kaohsiung 80708, Taiwan; yjjongnctu@gmail.com; 6 Genomics Research Center, Academia Sinica, Taipei 11574, Taiwan; winschen@gate.sinica.edu.tw; 7 Institute of Clinical Medicine, National Yang-Ming University, Taipei 11221, Taiwan

**Keywords:** Fabry disease, CRISPR, enzyme replacement therapy (ERT), drug screening, MG132

## Abstract

The CRISPR/Cas9 Genome-editing system has revealed promising potential for generating gene mutation, deletion, and correction in human cells. Application of this powerful tool in Fabry disease (FD), however, still needs to be explored. Enzyme replacement therapy (ERT), a regular administration of recombinant human α Gal A (rhα-GLA), is a currently available and effective treatment to clear the accumulated Gb3 in FD patients. However, the short half-life of rhα-GLA in human body limits its application. Moreover, lack of an appropriate in vitro disease model restricted the high-throughput screening of drugs for improving ERT efficacy. Therefore, it is worth establishing a large-expanded in vitro FD model for screening potential candidates, which can enhance and prolong ERT potency. Using CRISPR/Cas9-mediated gene knockout of *GLA* in HEK-293T cells, we generated GLA-null cells to investigate rhα-GLA cellular pharmacokinetics. The half-life of administrated rhα-GLA was around 24 h in GLA-null cells; co-administration of proteasome inhibitor MG132 and rhα-GLA significantly restored the GLA enzyme activity by two-fold compared with rhα-GLA alone. Furthermore, co-treatment of rhα-GLA/MG132 in patient-derived fibroblasts increased Gb3 clearance by 30%, compared with rhα-GLA treatment alone. Collectively, the CRISPR/Cas9-mediated GLA-knockout HEK-293T cells provide an in vitro FD model for evaluating the intracellular pharmacokinetics of the rhα-GLA as well as for screening candidates to prolong rhα-GLA potency. Using this model, we demonstrated that MG132 prolongs rhα-GLA half-life and enhanced Gb3 clearance, shedding light on the direction of enhancing ERT efficacy in FD treatment.

## 1. Introduction

Fabry disease (FD, OMIM 301500) is an inherited X-linked lysosomal storage disease (LSD) caused by mutations in the *GLA* gene that encodes α-galactosidase A (α-Gal A). Loss-of-function mutation in α-Gal A leads to progressive accumulation of globotriaosylceramide (Gb3) which contributes to decreased life expectancy [1,2,3]. There is so far no treatment to cure FD, but only supportive enzyme replacement therapies (ERTs) involving infusions of recombinant human α-Gal A (rhα-GLA), commercially named Fabrazyme (Agalsidase beta) and Replagal (Agalsidase alfa), to consistently stabilize patients’ kidney function, decrease neuropathic pain, and reverse or improve hypertrophic cardiomyopathy [4,5]. However, under body temperature and pH, the rhα-GLAs are unstable with shortened half-life of enzyme activity in vivo [4]. In addition, a number of issues including financial aspects [6] and generation of host antibodies against the therapeutic enzyme [7] have arisen from ERT-treated cases. These concerns limit the treatment efficacy and affect tolerability of rhα-GLA. Therefore, an alternative or combined therapy that reduces the cost or enhances ERT efficacy is urgent.

Recently, several pharmacological chaperones (PCs), small molecules designed for selective binding and stabilizing their target protein, have been identified as therapeutic for lysosomal storage disorders like FD [8,9,10]. Administration of the selective PCs to the mutated α-Gal A, particularly the missense mutants [11,12], facilitates the mutated α-Gal A to pass the protein quality control system in endoplasmic reticulum (ER) and potentiates their folding, maturation and/or cellular trafficking, hence resulting in effective lysosomal delivery of α-Gal A [13,14,15,16]. In addition, the selective PC was reported to enhance the ERT efficacy in vivo by prolonging rhα-GLA stability and reducing rhα-GLA degradation [17,18]. These results suggested that the proteostasis network, which consists of pathways that influence protein synthesis, folding, trafficking, disaggregation and degradation in cells, plays an important role in ERT efficacy. In order to broaden the therapeutic strategy for FD, the synergistic effects of the proteostasis modulators in combining with rhα-GLA treatment should be systematically evaluated in a high-throughput manner.

To date, the FD model is dependent on GLA knockout (KO) mice [19] or FD patient-derived fibroblasts [20]. However, neither model is suitable for high-throughput drug screening and easily available for study. Currently, CRISPR/Cas9 emerges as a powerful genome-editing technique providing the opportunity to delete genes in human cells efficiently [21,22,23]. Therefore, it is feasible to generate GLA-KO human cell lines by a CRISPR/Cas9-mediated Genome-editing method for screening the candidate molecules to improve ERT efficacy. In the present study, we applied CRISPR/Cas9 technique to establish GLA-KO human cell lines to evaluate the efficacy of the proteostasis modulator, i.e., MG132, on potentiating the rhα-GLA activity. Administration of MG132 enhanced intracellular half-life of the rhα-GLA in the GLA-KO cells. Moreover, MG132 potentiated the rhα-GLA-mediated Gb3 clearance in FD patient-isolated fibroblasts, thus shedding light on improving the ERT efficacy with proteostasis modulator co-treatment for FD patients. Collectedly, the CRISPR/Cas9-mediated GLA-KO cells will be a potential FD cell model for high-throughput screening of drug candidates that prolong rhα-GLA potency.

## 2. Results

### 2.1. CRISPR/Cas9-Mediated Gene Editing of GLA Effectively and Completely Ablated Endogenous GLA Protein Expression in Human Cells

**Figure 1:**
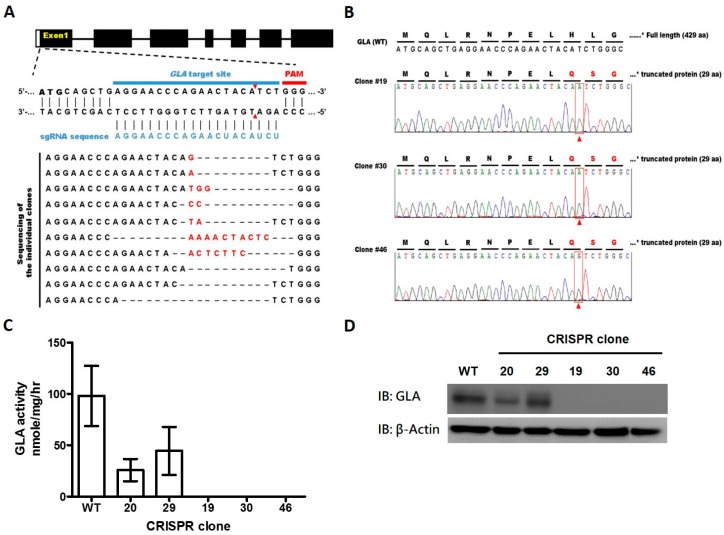
CRISPR/Cas9-mediated genome disruption of human *GLA* results in cell lacking detectable GLA expression. (**A**) The sgRNA sequence for *GLA* gene targeting and the mutation spectra of *GLA* gene was illustrated. The sgRNA sequence (5′-AGGAACCCAGAACUACAUCU-3′) was labeled as blue. Sequence within the blue bar- and red bar-labeled range indicated the *GLA* target site of sgRNA and the upstream protospacer adjacent motif (PAM), respectively. The double-strand breaking site was indicated as the red arrowheads. The indel spectrum of each clone was shown in alignment, in addition, the black texts, red texts, and dash represented the original *GLA* sequence, inserted nucleotide, and deleted nucleotide/alignment space, respectively in the *GLA* target site; (**B**) Sequence analysis confirmed the mutations with one nucleotide insertion (indicated by red arrowhead in red square) in the selected GLA-null cell lines, i.e., clone 19, 30, and 46, in comparison with the wild-type (WT) human *GLA* sequence. The GLA enzyme activities (**C**) and the GLA protein expressions (**D**) were determined in the parental HEK-293T cells and the selected GLA-null cell lines.

In order to disrupt GLA expression in HEK-293T cells, the GLA-specific single-guide RNA (sgRNA) was designed in conjunction with the “Optimized CRISPR Design (available online: http://crispr.mit.edu/)” from Zhang Lab. The sgRNA sequence and the target site are shown in Figure 1A. Following the plasmid preparation, HEK-293T cells were transfected with the GLA-specific sgRNA/Cas9 dual expression vector, pSpCas9 (BB)-2A-GFP, by electroporation. There was then a DNA double-stranded break at the sgRNA targeting sequence by Cas9 protein which represents GFP signaling. Fluorsence-activated cell sorting (FACS) was then used to enrich gene-edited cell population and seeding in a 10 cm dish. Thus, single cell-derived stable clones were established. The GLA expression and enzyme activity were barely detected in the 21 stable clones out of 23 (Figure S1A,B). Subsequently, the genetic lesion caused by GLA-targeted CRISPR/Cas9 in the selected clones were identified by Sanger sequencing (Figure 1A). There are several clones that have demonstrated the changes at the target region in the chromosome. Three GLA-deficient stable clones (clones #19, 30 and 46) were selected for the following study because each has one nucleotide insertion: an adenine insertion in clone 19 and 30, and a guanine insertion in clone 46, respectively (Figure 1B). It has been noted that the Indel (insertion & deletion) spectra of clones #19, 30 and 46 are homozygous one nucleotide insertion. The insertions resulted in a frame-shift in GLA coding sequence that caused truncation of GLA and led to complete loss of GLA enzyme activity. To further confirm the CRISPR/Cas9-mediated GLA knockout, the three selected clones were subjected to GLA enzyme activity assay (Figure 1C) and Western blot against GLA antibody (Figure 1D); no enzyme activity and protein expression of GLA were detected in clone 19, 30, and 46 compared to wild-type HEK-293T cells. It has to be noted that there were no significant differences in cell proliferation rate between the three clones of GLA-null cells and the wild-type HEK-293T cells (Figure S1C). Therefore, by conducting the CRISPR/Cas9 genome-editing technique, we successfully established the GLA-null cell lines with normal cell proliferation and complete deficiency of the endogenous GLA enzyme activity; these cell lines provided a potential platform to determine the intracellular pharmacokinetics of the exogenous administrated rhα-GLA.

### 2.2. HEK-293T GLA-Null Cell Lines Serve as a Platform for rhα-GLA Intracellular Pharmacokinetics Assay

**Figure 2:**
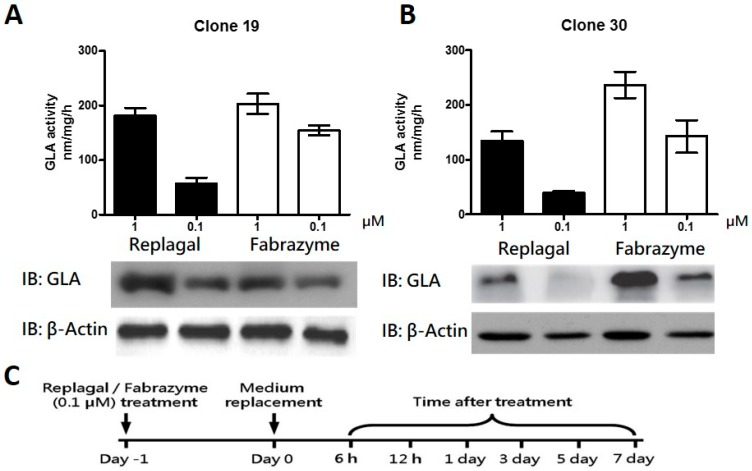

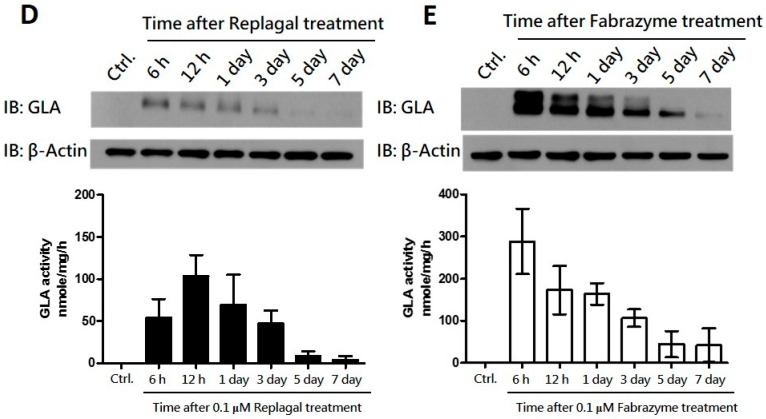
Enzyme activity and protein expression of rhα-GLA in the GLA-null cell lines. The CRISPR/Cas9-mediated GLA-null HEK-293T cell lines, i.e., clone 19 (**A**) and clone 30 (**B**) were incubated with 0.1 and 1 µM Replagal or Fabrazyme for 6 h. The GLA enzyme activities were measured based on the description of “Materials and Methods” 1 day after rhα-GLA treatment and the results were presented as mean ± SD (*n* ≥ 3). In addition, the rhα-GLA protein expression in cell lysates was determined by immunoblot probing of the monoclonal antibody against the human GLA. β-actin was used as loading control. Representative results out of three independent experiments are presented; (**C**) Time schedule determining the intracellular pharmacokinetics of rhα-GLA was illustrated. Briefly, the GLA-null cell lines were treated with 0.1 µM Replagal (**D**) or Fabrazyme (**E**) for 24 h. Following the treatment, the rhα-GLA was replaced with fresh medium and the intracellular rhα-GLA protein levels and the corresponding GLA enzyme activity were measured accordingly at the indicated time (from 6 h to 7 days). β-actin was used as loading control in the immunoblot assay. Representative immunoblot data are presented. In addition, the GLA enzyme activities are presented as mean ± SD (*n* ≥ 3).

The GLA enzyme activity and its protein level were determined in the GLA-null clone 19 (Figure 2A) and clone 30 (Figure 2B) following the treatment with rhα-GLA (Replagal and Fabrazyme). Results of the Western blot and GLA enzyme activity assay showed that exogenous administration of rhα-GLA was clearly detected in clone 19 and 30 in a dose-dependent manner. These results suggested no significant differences in rhα-GLA activity and expression between different GLA-null cell-lines. We then determined the intracellular pharmacokinetics of rhα-GLA in the GLA-null clone 30 with a schedule as shown in Figure 2C. The exogenous administrated rhα-GLA was decreased in a time-dependent manner in the GLA-null cells treated with Replagal (Figure 2D) and Fabrazyme (Figure 2E). It is worthwhile to note that the rhα-GLA uptake and GLA activity of Replagal were obviously lower than that of Fabrazyme in the GLA-null cells. These differences may simply attribute to the different glycosylation between Fabrazyme and Replagal because a previous study has observed Fabrazyme having higher levels of phosphorylated oligomannose residues and more abundant uptake into Fabry fibroblasts in vitro, in comparison with Replagal [24]. Overall, these results indicated that Replagal and Fabrazyme could be detected within effective activity in cells one day after treatment, and gradually eliminated from cells within the following time (from 6 h to 7 days after treatment). In addition, the rhα-GLA protein levels and its enzyme activity were attenuated to nearly 50% within 3 days, which suggested the half-life of the exogenous rhα-GLA may sustain for 24 to 72 h after treatment in the cell. Furthermore, the intracellular rhα-GLA was barely detectable 7 days after either Replagal or Fabrazyme treatment. These results indicated that the GLA-null cells are capable of the rhα-GLA intracellular pharmacokinetics assay without the interference of endogenous GLA protein/enzyme activity. Therefore, our established GLA-null cells can be used as an in vitro cell model for screening potential drugs that prolong the rhα-GLA half-life and improve its enzyme activity specifically.

### 2.3. Co-Administration of MG132 Improved the Stability and Activity of rhα-GLA in the GLA-Null Cell Line without Cytotoxic Effects

**Figure 3:**
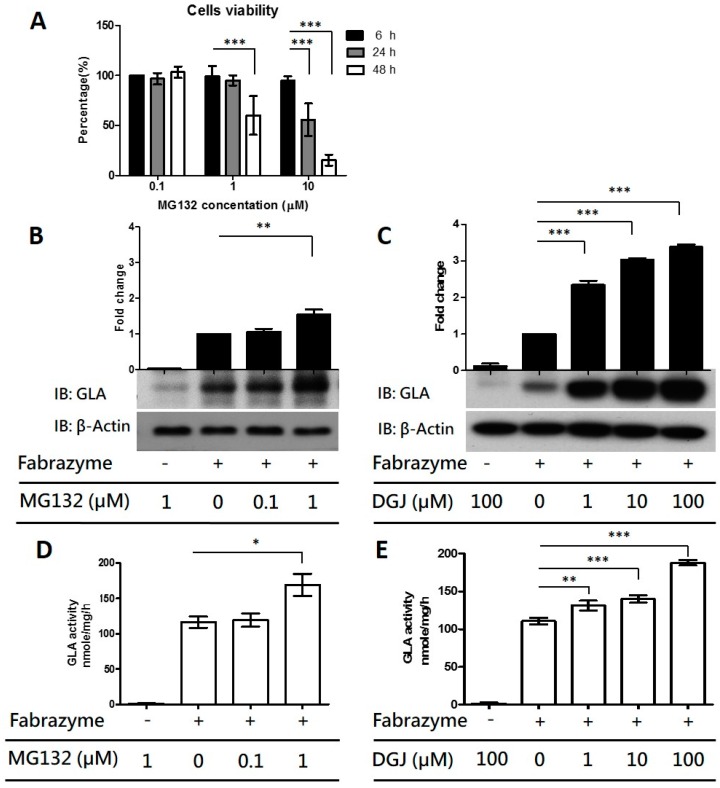
Co-administration of rhα-GLA and MG132 increases the enzyme activity and protein stability of rhα-GLA in vitro. (**A**) Cytotoxicity of proteasome inhibitors MG132 in the GLA-null cells were determined by WST-8 assay followed as the description in “Materials and Methods”. Briefly, the cells were treated with MG132 (0.1, 1, and 10 µM) for 6, 24, and 48 h. The cell viability of each treatment was determined by comparing with the vehicle control. The data are presented as mean ± SD (*n* ≥ 3). *** *p* < 0.001 vs. the 6 h post-treatment; (**B**) Following co-treatment of MG132 (0.1 and 1 µM) with or without 0.1 µM Fabrazyme for 24 h, the medium was refreshed, and the cells were cultured for another 24 h. Protein levels of rhα-GLA were determined by using immunoblot analysis with β-actin as loading control. Representative immunoblot data are presented. Relative levels of rhα-GLA protein were quantified by densitometry scanning; results are presented as mean ± SD (*n* = 3). ** *p* < 0.01 vs. the treatment controls (MG132 = 0 µM); (**C**) In following the treatment schedule, the GLA-null cells co-treated with 1-deoxygalactonojirimycin (DGJ) (1, 10, and 100 µM), with or without 0.1 µM Fabrazyme, were collected for immunoblot analysis of the rhα-GLA protein levels. Representative immunoblot data are presented, and the quantitative results are presented as mean ± SD (*n* = 3). *** *p* < 0.001 vs. the treatment controls (DGJ = 0 µM). The corresponding GLA enzyme activity in the cells co-treated with MG132 (**D**) and DGJ (**E**) was determined respectively. Data are presented as means ± SD (*n* ≥ 3). * *p* < 0.05, ** *p* < 0.01, and *** *p* < 0.001 vs. the treatment controls (MG132 or DGJ = 0 µM, respectively). ”+” and “-“ represented the cell treatment with and without Fabrazyme administration, respectively.

As shown in Figure 2D,E, the exogenous rhα-GLA was eliminated from GLA-null cells within one week. Proteostasis networks including macro-autophagy and ubiquitin-proteasome pathways that are responsible for protein homeostasis may be involved in the clearance of the rhα-GLA from cells. However, the autophagy flux is impaired in patients with Fabry disease [25], hence, the ubiquitin-proteasome pathway may be the potential candidate to mediate the elimination of exogenous rhα-GLA. To evaluate this notion, the influences of proteasome inhibitor MG132 on rhα-GLA stability have been determined in GLA-null cells. Because proteasome inhibitor could suppress cell growth and induce apoptosis in various cells [26], the cytotoxic effect of MG132 was firstly examined on the GLA-null cell line. As shown in Figure 3A, GLA-null cells treated with 1 µM MG132 showed no significant difference in cell viability within 24 h. However, only 50% of cells survived 48 h after MG132 treatment. In addition, the cell survival rates are significantly decreased with 10 μM MG132 treatment. Therefore, the minor lethal dosage of 1 µM MG132 following 24 h treatment was applied to determine the influences of proteostasis networks on rhα-GLA stability. As shown in Figure 2, the intracellular pharmacokinetics of Replagal and Fabrazyme are comparable in the GLA-null cells. However, the uptake ratio of rhα-GLA in cells for Fabrazyme treatment is significantly higher than that with Replagal treatment. In order to maximize the possible therapeutic effect of ERT, Fabrazyme was used for the following studies. Co-treatment of 1 µM MG132 with Fabrazyme for 24 h in the GLA-null cells showed two-fold higher intracellular rhα-GLA protein levels than cells treated with Fabrazyme only (Figure 3B). In addition, cells with rhα-GLA and MG132 (1 µM) co-treatment resulted in 20% greater GLA enzyme activity compared to rhα-GLA treatment only (Figure 3D). The 1-deoxygalactonojirimycin (DGJ), a pharmacological chaperone, served as a positive control to evaluate the sensitivity of our established GLA-null cells as the platform for screening of the potential drug candidates in maintenance of the intracellular rhα-GLA stability. Previous studies have indicated that 10–100 µM DGJ is sufficient for sustaining the rhα-GLA activity [18,20] As shown in Figure 3C,E, cells co-treated with DGJ significantly increased the rhα-GLA protein levels and the GLA enzyme activity, respectively, in a dose-dependent manner. Collectively, these results suggested that the GLA-null cells are feasible and capable of screening potential drug candidates for maintaining intracellular rhα-GLA stability. Our data showed that MG132 could be one of the potential molecules in sustaining the intracellular stability and activity of rhα-GLA.

### 2.4. MG132 Maintained Intracellular Amount of rhα-GLA and Reduced Gb3 Accumulation in Fabry Patient-Derived Fibroblasts

**Figure 4:**
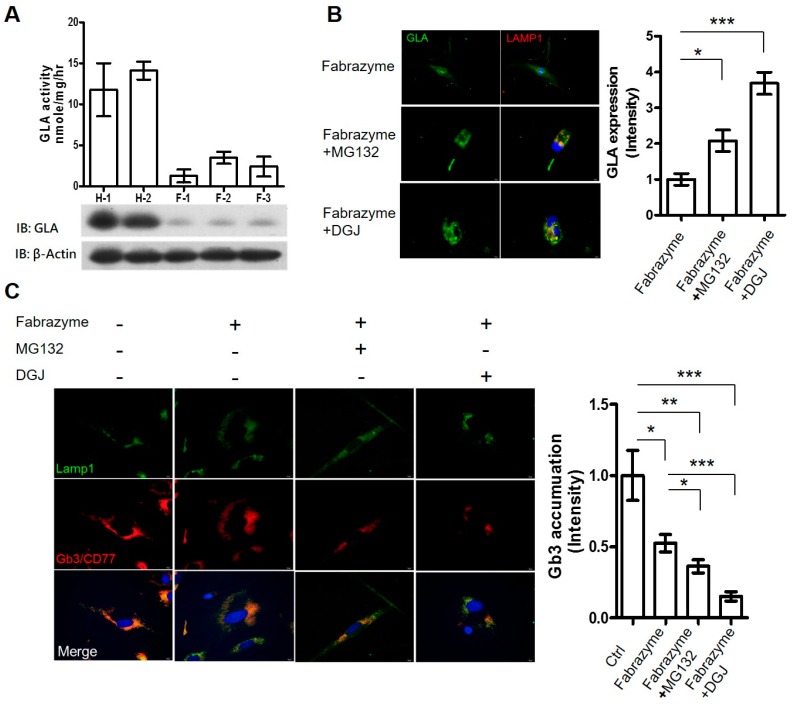
Subcellular localization of the rhα-GLA and the clearance of the lysosomal-accumulated Gb3 in Fabry patient-derived fibroblasts co-treated with Replagal and MG132. (**A**) The protein expressions and enzyme activities of GLA were characterized in the fibroblasts that derived from healthy subjects (H-1 and H-2), and the FD patient carrying *GLA* IVS4 + 919G>A mutation (F-1, F-2, and F-3); (**B**) Subcellular localization of the Fabrazyme rhα-GLA was determined by immunofluorescence staining in the FD patient fibroblast co-treatment of 0.1 µM Fabrazyme with 1 µM MG132 or 100 µM DGJ. The rhα-GLA was probed with anti-GLA antibody and then labeled with FITC (green). The lysosome marker LAMP1 was probed with rabbit anti-LAMP1 antibody and then labeled with TRITC (red). Cell nuclei were labeled with DAPI (blue). A representative immunofluorescence analysis from three independent experiments is shown. The quantitative results are presented as mean ± SEM (*n* ≥ 10 cells). * *p* < 0.05 and *** *p* < 0.001 vs. Fabrazyme treatment only. Scale bar equals 20 µm; (**C**) Lysosomal-accumulated Gb3 was determined by immunofluorescence staining. The FD patient-derived fibroblasts were grown on coverslips and co-treated with 0.1 µM Fabrazyme—1 µM MG132 or 100 µM DGJ for 24 h—and then replaced in fresh medium for another 10 days. Subsequently, the Gb3 was probed with rat anti-Gb3/CD77 antibody and then labeled with TRITC (red). The lysosome marker LAMP1 was probed with rabbit anti-LAMP1 antibody and then labeled with FITC (green). Cell nuclei were labeled with DAPI (blue). The color yellow in the merge indicated the lysosomal-accumulated Gb3. Representative immunofluorescence data are presented, and the quantitative results are presented as mean ± SEM (*n* ≥ 10). * *p* < 0.05, ** *p* < 0.01, and *** *p* < 0.001 vs. the non-treatment (Ctrl) or Fabrazyme treatment only. ”+” and “-“ represented the cell treatment with and without Fabrazyme/MG132/DGJ administration, respectively. Scale bar equals 20 µm.

In order to evaluate the role of MG132 on prolonging ERT efficacy, the effects of MG132 co-administrated with rhα-GLA were further accessed in FD patient-derived cells. The fibroblasts derived from FD patients with *GLA* IVS4 + 919G>A mutation—the most common type of GLA mutation in Taiwan—in addition to the alternative splicing that introduces a 57-nucleotide (nt) intronic sequence to the α-Gal A transcript from intron 4 of the gene, have been identified [27,28,29] as expressing a lower level of endogenous GLA enzyme activity and protein expression compared with the fibroblasts derived from healthy subjects (Figure 4A). The *GLA* IVS4 + 919G>A mutation was confirmed by RT-PCR and DNA sequencing (Figure S1D,E). Following co-treatment of MG132 (1 µM) or DGJ (100 µM) with 0.1 µM Fabrazyme for 24 h, the large amount of intracellular rhα-GLA was obviously co-localized with the Lamp1, a lysosome-specific marker, in immunofluorescence stain (Figure 4B). In comparison with Fabrazyme treatment only, the intracellular rhα-GLA intensity is increased by two-fold and 3.5-fold cells co-administrating with MG132/Fabrazyme and DGJ/Fabrazyme, respectively. GLA deficiency results in the progressive lysososmal accumulation of globotriaosylceramide (Gb3/CD77) in the tissue. Therefore, based on immunofluorescence staining of Fabry patients′ fibroblast with anti-CD77 antibody revealed that abundant Gb3 accumulation. However, in addition, the lysosomal Gb3 accumulations in the FD patient-derived fibroblasts were significantly reduced 10 days after incubation of MG132/Fabrazyme or DGI/Fabrazyme, compared with Fabrazyme treatment only (Figure 4C). The Gb3 accumulation in cells with MG132/Fabrazyme and DGJ/Fabrazyme treatment are 30% and 50% lower than that with Fabrazyme treatment only. These results indicated that co-administration of MG132 and rhα-GLA prolonged GLA activity and significantly reduced lysosomal Gb3 accumulation in the fibroblasts carrying *GLA* IVS4 + 919G>A mutation. Furthermore, these results suggested that MG132 may improve the intracellular stability and lysosomal delivery of rhα-GLA, and potentiate the lysosomal Gb3 clearance, similar to the function of the pharmacological chaperone DGJ to prolong the ERT efficacy.

## 3. Discussion

Previous studies have reported the application of the genome-editing technique in the human pluripotent stem cells to model diseases that were caused by single-gene mutations for drug discovery [30,31]. In the present study, we generated GLA-null HEK-293T cell lines by CRISPR/Cas9 genome-editing system. We demonstrated that targeting *GLA* with CRISPR/Cas9 resulted in the complete ablation of GLA protein expression with barely detectable GLA enzyme activity (Figure 1 and Figure 2). Unlike siRNA, which is transient and often presents different knockdown levels, the created GLA-null cells were stable, heritable, and a useful model to screen molecules that could improve rhα-GLA stability in a human cell without the interferences of endogenous GLA expression. In addition, the CRISPR/Cas9 genome-editing technique has also been used to develop human kidney cell model of FD in human immortalized podocytes [32]. These results, including our present report, elaborated the application of CRISPR/Cas9 in studying inherited diseases, e.g., FD.

The previous studies have conducted HEK-293 cells as the in vitro model for determining the responses of PCs on mutated GLAs [33,34,35]. These results suggested that the use of HEK-293 cells on screening or validating the drug efficacy for Fabry disease is feasible and applicable. However, the endogenous GLA expression in HEK-293T cells (Figure 1C,D) may hinder us from determining the exogenous rhα-GLA efficacy. Therefore, our established GLA-null cells can serve as a disease model with a clear background to investigate rhα-GLA cellular pharmacokinetics.

The rhα-GLA of Replagal and Fabrazyme are produced from human cell lines and cultured CHO cells, respectively, that show slight differences of the post-translational modification, particularly the glycosylation. The previous study identifying the proportion of mannose-6-phsophate in Fabrazyme is higher than that in Replagal, resulting in more abundant uptake of Fabrazyme into Fabry fibroblasts in vitro in comparison with Replagal [24]. However, the bio-distribution, pharmacokinetics, and the clinical responses between Fabrazyme and Replagal are similar in the animal studies in vivo and in the clinical trials [36,37,38]. These results suggested that rhα-GLA of Fabrazyme and Replagal are functionally identical in the enzyme activity and the protein turnover. In our present study, the intracellular pharmacokinetics of the rhα-GLA, i.e., Fabrazyme and Replagal, are consistent with the previous studies that the half-life of the administrated rhα-GLAs was around 24 h, and the rhα-GLA uptake of Fabrazyme was obviously higher than that of Replagal in the GLA-null cells (Figure 2D,E).

Long-term ERT has been reported to reduce the risk of complications, e.g., stroke and end-stage renal disease, development in FD patients [39], and to prevent the disease progression in patients who initiated treatment at a young age [40]. These results suggested the beneficial effects of rhα-GLA administration on FD treatment; however, the financial burdens [6] and the risk of immune responses against the rhα-GLA [7] restricted the application of ERT for FD patients. Therefore, development of novel therapeutic strategies is urgent for FD patients. Recently, pharmacological chaperone therapy has been considered as a novel treatment for lysosomal storage disorders including Fabry disease. The DGJ, one kind of iminosugar that acts as the galactose analog, has been reported to rescue the protein folding and activity of GLA in FD patients carrying missense mutations [11,15,41]. However, it has been reported that only 42% of *GLA* mutation types carried in FD patients benefited from PC treatment [42]. Collectively, a combined therapy that reduces the cost or enhances ERT efficacy may be adequate for most FD cases. Therefore, in the present study, we mainly focus on the development of the GLA-null cells for determining the influences of potential candidates on enhance or prolong the rhα-GLA potency.

By using our established GLA-null cells, co-treatment of DGJ significantly enhanced the intracellular GLA enzyme activity of Fabrazyme in a dose-dependent manner (Figure 3E) and sustained the rhα-GLA protein stability in comparison with the Fabrazyme treatment only (Figure 3C). Our results echo previous studies claiming that co-administration of the iminosugar-based PC and rhα-GLA lead to maintaining stability of rhα-GLA and improving the potency of ERT in a Fabry mouse model [17,18]. Overall, our established GLA-null cell lines could be a suitable in vitro cell model for screening the potential candidates that enhance or prolong the rhα-GLA potency.

To broaden the therapeutic strategy for FD, the influences of the proteasome inhibitor, i.e., MG132, on rhα-GLA potency was evaluated in our established GLA-null cells. MG132 has been reported to increase the protein stability and to restore the enzyme activity of the misfolding mutants in LSDs such as Niemann-Pick type C (NPC) disease [43], Pompe disease [44], and Sialidosis [45]. However, to our best knowledge, the influences of proteasome inhibitors on the ERT potency/efficacy for FD have not been determined. Co-administration of MG132 and rhα-GLA clearly maintained the protein stability and GLA enzyme activity of rhα-GLA (Figure 3B,D). In addition, MG132 treatment improved the rhα-GLA-mediated lysosomal Gb3 clearance in the FD patient-derived fibroblast (Figure 4) that further strengthen the beneficial effects of MG132 on ERT efficacy. A previous study has indicated that treatment of the proteasome inhibitors including MG132 cannot restore the enzyme activity of GLA with missense mutations [46]. The differences between our present study and that of Lukas et al. might simply be attributed to different concentrations of MG132 (0.1 µM MG132 by Lukas et al. in comparison to 1 μM MG132 in the present study). However, we cannot completely rule out the possibility that the exogenous rhα-GLA might go through different protein turnover pathways from the endogenous misfolding GLA.

In addition to the proteasome inhibition, MG132 has also been reported to induce heat shock protein (HSP) expression that protected the cardiomyocytes [47] and lens epithelia cells [48] from apoptosis. Furthermore, MG132 treatment enhanced cellular trafficking and enzyme activity of the misfolding mutated glucocerebrosidase through inducing the expression of HSPs and ER chaperones, i.e., Hsp40, Hsp70, Hsp90, and Bip in fibroblasts from Gaucher disease patients [49]. In the present study, we determined that the expression of Hsp60 and Hsp70 are induced following MG132 treatment in the GLA-null cells (Figure S2) implicating the involvement of cellular chaperones in MG132-mediated rhα-GLA maintenance. However, it has not yet been determined whether the proteasome activity plays a role in HSP expression. Recently, the Hsp70 has been shown to rescue the Niemann-Pick disease by stabilizing the lysosomes [50], thus suggesting that the potential therapeutic strategy of heat shock proteins for LSD treatment [51]. In the future, it will be worth addressing the influences of HSP expression and/or the heat shock responses on ERT potency/efficacy.

## 4. Materials and Methods

### 4.1. Cell Culture

Human fibroblasts were obtained from skin biopsies of patients with *GLA* IVS4 919G>A mutation or healthy subjects and were approved by the TVGH-IRB committee, and signed patient consent forms were obtained (IRB #: 2013-06-025B). The fibroblasts and HEK-293T cells were maintained in Dulbecco′s Modified Eagle Medium (DMEM) supplemented with 10% fetal bovine serum (Gibco, Carisbad, CA, USA) under humidified atmosphere of 5% CO_2_ at 37 °C. The rhα-GLA, i.e., Agalsidase-α (Replagal, Shire Pharmaceuticals, Lexington, MA, USA) and Agalsidase-β (Fabrazyme, Genzyme Corporation, Cambridge, MA, USA), was diluted with culture medium for cell treatment in the indicated time. MG132 and 1-deoxygalactonojirimycin DGJ was dissolved in dimethyl sulfoxide (DMSO; Sigma-Aldrich, St. Louis, MO, USA) solution buffer.

### 4.2. CRISPR/Cas9 Plasmid Construction and Transfection

The CRISPR/Cas9 with T2A-eGFP co-expression vector pSpCas9 (BB)–2A-GFP (pX-458) was purchased from Addgene. The exon 1 of *GLA* was selected for guiding RNA design and sequence (5′-AGGAACCCAGAACTACATCT-3′) was cloned into pX-458 (abbreviated as GLA-Cas9-GFP) as previously described [52]. The GLA-specific targeting plasmid was transfected into HEK-293T cells by electroporation using Nucleofector™ System (Lonza, Basel, Switzerland) following the manufacturer′s protocol. Briefly, HEK-293T cells were cultured to 80%–90% confluence, then harvested and washed with 1× phosphate buffered saline (PBS) without Ca^2+^ and Mg^2+^. Approximately 8 × 10^5^ cell pellets were resuspended in the pre-mixture solution with 2 µg GLA-Cas9-GFP, and the optimized protocol was used for electroporation.

### 4.3. Analysis of CRISPR/Cas9-Mediated Indel Formation in GLA Gene

To determine the Indel spectra in GLA gene, the genomic DNA were extracted and used for PCR amplification of target sites with the primer pair 5′-CACACACCAACCTCTAACGATACC-3′ (forward) and 5′-CCAGGAAAGGTCACACAGAGAAAG-3′ (reversed). PCR products were TA cloned into the pGEM-T Easy vector (Promega, Madison, WI, USA). Subsequently, 20 colonies were sequenced using T7 forward primer for each cell line. ContigExpress of Vector NTI was used to align the results of sequence and determine the indel spectra in GLA target site.

### 4.4. Immunofluorescence Stain

Cells were fixed with 1% paraformaldehyde solution at room temperature (RT) for 15 min and permeabilized with 0.1% Triton X-100 for 10 min. After several washes with 1× PBS, fixed cells were blocked with 3% BSA and 5% FBS at RT for 1 h and incubated with rat mAb against Gb3/CD77 (1:100, ab19795, Abcam, Cambridge, UK) or rabbit mAb against LAMP1 (1:200, #9091, Cell Signaling, Beverly, MA, USA) overnight at 4 °C. Cells were washed three times with 1× PBS and incubated with secondary antibodies at RT for 1 h. Finally, the cells were mounted and observed using a fluorescent or FV10i confocal microscope (Olympus, Center Valley, PA, USA).

### 4.5. Western Blotting Analysis

Cells were lysed with RIPA lysis buffer (0.5 M Tris-HCl, pH 7.4, 1.5 M NaCl, 2.5% deoxycholic acid, 10% NP-40, 10 mM EDTA, protease inhibitor), and protein concentrations were measured by the Bradford method. The proteins were subjected to SDS-PAGE and then transferred to PVDF membrane. Membranes were blocked with 5% non-fat milk and probed with monoclonal antibodies against α-galactosidase A (GeneTex, San Antonio, TX, USA), β-actin (Sigma-Aldrich, St. Louis, MO, USA), respectively. Immunoreactive bands were visualized by chemoluminescence detection reagents (Millipore, Bethesda, MA, USA) and detected using an X-ray film. β-actin was served as the loading control.

### 4.6. GLA Enzyme Activity

Cells were washed twice with 1× PBS and were lysed in 60 µL lysis buffer (27 mM sodium citrate, 46 mM sodium phosphate dibasic, 0.5% Triton X-100). The amount of 10 µL cell lysate was added to 50 µL assay buffer containing 6 mM 4-methylumbelliferyl-α-d-galactopyranoside and 117 mM *N*-acetyl-d-galactosamine and incubated at 37 °C for 1 h. The 4-methylumbelliferone dissolved in methanol was used as a standard ranging from 0.15 to 5000 µM. After that, 70 µL glycine-carbonate solution (pH 10.8) was then added to stop the reaction, and fluorescence was detected by the microplate reader (em/ex = 365/448 nm). The enzyme activity was normalized by protein concentration of cell lysate.

### 4.7. Cell Viability Assay

Briefly, HEK-293T cells were plated in a 96-well plate at a cell density of 5 × 10^3^ cells/well. After 24 h, cells were treated with indicated concentration of MG132. Cell viability was measured in the indicated time by WST-8 assay (Dojindo, Kumamoto, Japan).

### 4.8. Statistical Analysis

ImageJ (gel analyzer plugin) was used for quantification of Western blots and immunostaining, and data were presented as mean ± SD (or 9SEM) by GraphPad Prism 5 (GraphPad Prism Software version 5.01). Statistical significance was assessed by unpaired Student′s *t* test or one-way analysis of variance (ANOVA) with post hoc analysis using Tukey′s multiple comparison test for parametric data. A *p*-value less than 0.05 will be defined as statistically significant.

## 5. Conclusions

To summarize, the present study established that the human cell lines with CRISPR/Cas9-mediated genetic disruption of GLA are devoid of both detectable GLA protein expression and enzyme activity for evaluating the rhα-GLA stability (Figure 1 and Figure 2). In addition, this in vitro Fabry disease (FD) model provides a precedent for the genesis of other GLA-null human cells from different cell types including primary human stem cells. Furthermore, we identified that MG132 could prolong rhα-GLA activity in the GLA-null cell lines (Figure 3) and improve the ERT potency in the FD patient-derived fibroblast (Figure 4). These experiments described here provide a cell model that can be used to evaluate rhα-GLA activity and stability without the interference of endogenous GLA expression. In considering the sustainability of a patient′s primary cells, it is convincing that a relatively immortalized cell line, i.e., HEK-293T cells, would be an easy-to-operate source for incorporation into high-throughput screening system (HTS). Furthermore, both spectrometry-based GLA activity assay and visualized Gb3 immunostaining can be properly modified under the principle of HTS analysis. In order to identify the potential candidates, which can enhance or prolong the ERT potency/efficacy, the GLA-null cell lines should be applied as a first step because the cell lines are more flexible for optimization of the treatment regimes in drug screening. Subsequently, the patient-specific cells, e.g., the fibroblasts derived from patients with FD, could be applied as a second step to validate the beneficial effects of the selected drugs on FD. Overall, the CRISPR/Cas9-mediated GLA-KO cells will be an appropriate in vitro FD cell model for HTS of drug candidates that prolong the rhα-GLA potency in the future.

## Supplementary Materials

Supplementary materials can be found at www.mdpi.com/1422-0067/17/12/2089/s1.

## Abbreviations

CRISPR	Clustered regularly interspaced short palindromic repeats
sgRNA	single-guide RNA
Cas9	CRISPR-associated protein
DGJ	1-deoxygalactonojirimycin
ERT	enzyme replacement therapy
FD	Fabry disease
Gb3	globotriaosylceramide
α-Gal A	α-galactosidase A
KO	knockout
HEK-293T	human embryonic kidney 293T cells
HSP	heat shock protein
LSD	lysosomal storage disease
PC	pharmacological chaperone
rhα-GLA	recombinant human α Gal A
