# Supplementary material for: Using CRISPR/Cas9-Mediated GLA Gene Knockout as an In Vitro Drug Screening Model for Fabry Disease
Source: Int J Mol Sci. 2016 Dec 13;17(12):2089. doi: 10.3390/ijms17122089 (PMC5187889; doi:10.3390/ijms17122089)
Supplement: Supplementary file 1 [file ijms-17-02089-s001.pdf]

# Supplementary Materials: Using CRISPR/Cas9-Mediated *GLA* Gene Knockout as an In Vitro Drug Screening Model for Fabry Disease

**Hui-Yung Song, Huai-Chih Chiang, Wei-Lien Tseng, Ping Wu, Chian-Shiu Chien, Hsin-Bang Leu, Yi-Ping Yang, Mong-Lien Wang, Yuh-Jyh Jong, Chung-Hsuan Chen, Wen-Chung Yu and Shih-Hwa Chiou**

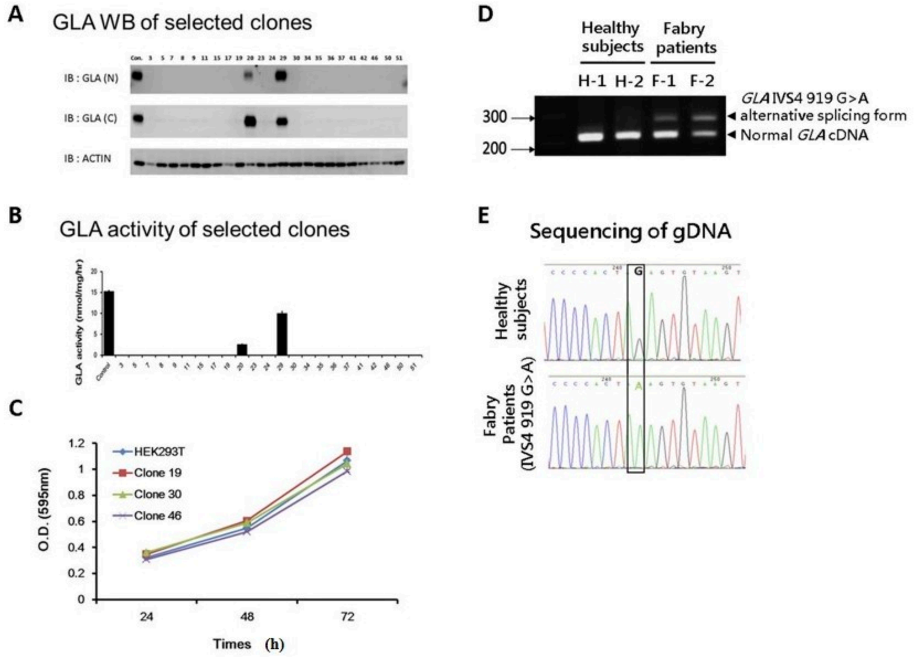


**Figure S1.** The GLA protein expression levels (**A**) and the GLA enzyme activity (**B**) were determined in 23-selected CRISPR/Cas9-mediated *GLA* knockout clones in HEK-293T cells; (**C**) Cell proliferation was determined in the parental HEK-293T cells and the GLA-null cell clone 19, 30 and 46; (**D**) The genotype analysis of the *GLA* cDNA at the junction between exon 4 and 5 was examined by RT-PCR amplification of 0.1 mg of total RNA that prepared from the FD patient-derived fibroblasts and from the healthy subject-derived fibroblasts. The primers 5′-GTCCTTGGCCCTGAATAG-3′ and 5′-GTCCAGCAACATCAACAATT-3′ were used for PCR, and then the results were analyzed by agarose gel electrophoresis; (**E**) Sequence analysis confirmed the nucleotide of GLA IVS4 + 919 is "G" (highlight in the black square) in the fibroblasts derived from healthy subjects comparing to "A" in the fibroblasts derived from patients with FD > A mutation in the FD patient-derived fibroblasts (represented as IVS4 919 G > A mutant).

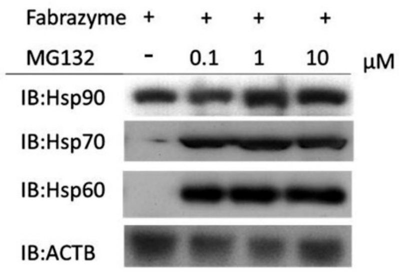


**Figure S2.** Protein expression levels of Hsp90, Hsp70, and Hsp60 were determined by immunoblot analysis in the GLA-null cells co-treating 0.1 μM Fabrazyme with MG132 (0.1, 1, and 10 μM) for 24 h. β-actin (ACTB) was used as loading control in the immunoblot assay. Representative immunoblot data are presented. ″+″ and "-" represented the cell treatment with and without Fabrazyme/MG132 administration, respectively.
